# DICER1 platform domain missense variants inhibit miRNA biogenesis and lead to tumor susceptibility

**DOI:** 10.1093/narcan/zcad030

**Published:** 2023-06-16

**Authors:** Dylan Pelletier, Anne-Laure Chong, Mona Wu, Leora Witkowski, Sophie Albert, Nelly Sabbaghian, Marc R Fabian, William D Foulkes

**Affiliations:** Department of Human Genetics, Medicine, McGill University, Montreal, QC, Canada; Cancer Axis, Lady Davis Institute, Jewish General Hospital, Montreal, QC, Canada; Faculty of Medicine, Université de Montréal, Montreal, QC, Canada; Department of Human Genetics, Medicine, McGill University, Montreal, QC, Canada; Cancer Axis, Lady Davis Institute, Jewish General Hospital, Montreal, QC, Canada; Department of Human Genetics, Medicine, McGill University, Montreal, QC, Canada; Cancer Axis, Lady Davis Institute, Jewish General Hospital, Montreal, QC, Canada; Department of Human Genetics, Medicine, McGill University, Montreal, QC, Canada; Cancer Axis, Lady Davis Institute, Jewish General Hospital, Montreal, QC, Canada; Department of Human Genetics, Medicine, McGill University, Montreal, QC, Canada; Cancer Axis, Lady Davis Institute, Jewish General Hospital, Montreal, QC, Canada; Cancer Axis, Lady Davis Institute, Jewish General Hospital, Montreal, QC, Canada; Gerald Bronfman Department of Oncology, McGill University, Montreal, QC, Canada; Division of Experimental Medicine, McGill University, Montreal, QC, Canada; Department of Biochemistry, McGill University, Montreal, QC, Canada; Department of Human Genetics, Medicine, McGill University, Montreal, QC, Canada; Cancer Axis, Lady Davis Institute, Jewish General Hospital, Montreal, QC, Canada; Gerald Bronfman Department of Oncology, McGill University, Montreal, QC, Canada; Cancer Research Program, Research Institute of the McGill University Health Centre, Montreal, QC, Canada

## Abstract

The endoribonuclease DICER1 plays an essential role in the microRNA (miRNA) biogenesis pathway, cleaving precursor miRNA (pre-miRNA) stem-loops to generate mature single-stranded miRNAs. Germline pathogenic variants (GPVs) in *DICER1* result in DICER1 tumor predisposition syndrome (DTPS), a mainly childhood-onset tumor susceptibility disorder. Most DTPS-causing GPVs are nonsense or frameshifting, with tumor development requiring a second somatic missense hit that impairs the DICER1 RNase IIIb domain. Interestingly, germline *DICER1* missense variants that cluster in the DICER1 Platform domain have been identified in some persons affected by tumors that also associate with DTPS. Here, we demonstrate that four of these Platform domain variants prevent DICER1 from producing mature miRNAs and as a result impair miRNA-mediated gene silencing. Importantly, we show that in contrast to canonical somatic missense variants that alter DICER1 cleavage activity, DICER1 proteins harboring these Platform variants fail to bind to pre-miRNA stem-loops. Taken together, this work sheds light upon a unique subset of GPVs causing DTPS and provides new insights into how alterations in the DICER1 Platform domain can impact miRNA biogenesis.

## Graphical Abstract

**Graphical Abstract:**
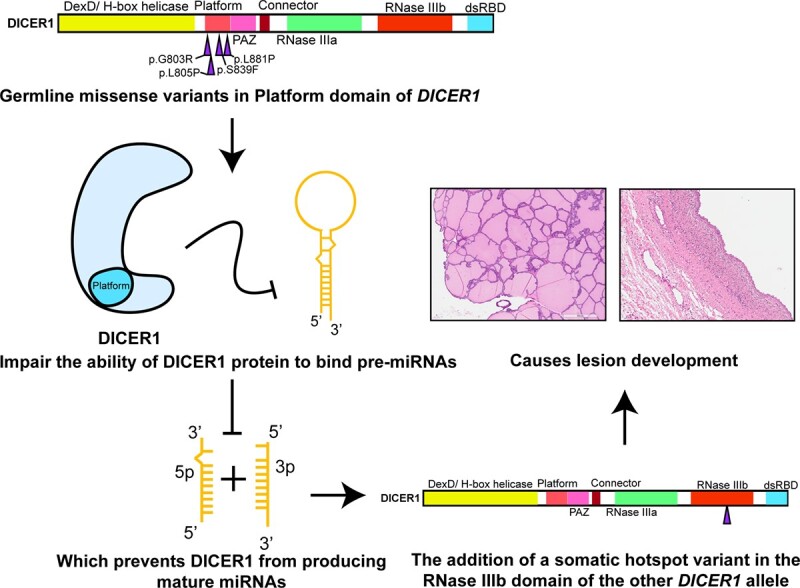

## INTRODUCTION

Micro (mi)RNAs are a class of small single-stranded RNAs essential for post-transcriptionally regulating gene expression. Mature miRNAs are generated in part by the DICER1 protein, a ribonuclease III (RNase III) enzyme that cleaves precursor (pre)-miRNA stem-loops using its RNase IIIa and IIIb domains (1–3). DICER1 contains seven structured domains with known functions (4). These include a N-terminal DExD/H-box helicase domain that plays an important role in substrate recognition (5). Following the DExD/H-box domain are Platform and Piwi/Argonaute/Zwille (PAZ) domains, thought to be important in binding to the 5′ phosphate and the 3′ end of pre-miRNAs, respectively (2,6–8). The connector helix is found between the PAZ and RNase IIIa domains. This helix plays an important role in providing distance between domains involved in pre-miRNA binding (Platform and PAZ) and pre-miRNA cleavage (RNase IIIa and IIIb). As a result, this helix allows DICER1 to cleave pre-miRNA at around 22 nucleotides from both its 3′ and 5′ ends (9,10,1,2) (6,7). This cleavage event is catalyzed by the RNase IIIa and RNase IIIb domains (1,3). These domains form a heterodimer around the pre-miRNA stem to mediate two cleavage events catalyzed by ion binding residues using magnesium or manganese as a co-factor. Dicer RNase IIIa and RNase IIIb domains cleave pre-miRNA stem-loops to generate mature 3′ arm (3p) and 5′ arm (5p) miRNAs, respectively (1,3). Finally, the C-terminus of the protein contains a double-stranded RNA-binding domain (dsRBD) that plays a role in pre-miRNA binding and clamping the RNA in the Dicer catalytic sites (5,11).

DICER1 tumor predisposition syndrome (DTPS) is a genetic disorder that predisposes affected individuals to a wide range of tumors that have mainly pediatric onset (12–14). These lesions include non-toxic multinodular goiter (MNG), pleuropulmonary blastoma (PPB), cystic nephroma (CN), Sertoli-Leydig cell tumor (SLCT) of the ovaries, sarcomas arising at several sites, and occasionally Wilms tumor (12–14). An individual is predisposed to such lesions if they harbor a germline pathogenic variant (GPV) in *DICER1*. This variant is usually a nonsense or frameshift variant which is predicted to produce a truncated protein (12). Tumorigenesis then requires a second somatic variant (second hit) affecting one of two exons that encode the RNase IIIb domain of *DICER1* in the originating tissue (15). Interestingly, a small subset of patients with DTPS-related clinical manifestations bear a germline *DICER1* missense variant (12), several of which fall into exons coding for the DICER1 Platform and PAZ domains. It can be difficult to determine if these variants are the cause of the syndromic presentations. This is because for tumor suppressor genes where the main pathogenic mechanism is biallelic inactivation of the causal gene in the associated tumors, it is expected that most causal variants will be predicted to truncate the protein. In this situation, the effect of missense variants on protein function is difficult to predict and therefore these variants are problematic for clinicians to interpret, leading to challenges to appropriate patient management (16,17).

Here, we utilized several *in vitro* biochemical and cell-based assays to determine if an array of germline missense variants that cluster in the DICER1 Platform domain impact DICER1 activity in such a way that is consistent with a causal role in DTPS.

## MATERIALS AND METHODS

### Plasmids

For generation of DICER1 mutant plasmids, pQCXIB-1xFLAG-DICER1 was used as a template for site-directed mutagenesis (SDM) using overlapping primers. For generation of *Renilla* miRNA sensor plasmids, we cloned the new target sites by digesting the pCl-RL-6xWT plasmid by conventional molecular cloning techniques using Not1 and Xba1 restriction enzymes (ThermoFisher Scientific). We then ligated the cut plasmid by using overlapping oligos as the insert.

### Antibodies

Antibody against DICER1 was purchased from Bethyl, against beta-tubulin from Millipore-Sigma, against GAPDH from Cell-Signalling, against hnRNP A1 from Abcam. All antibodies were used at a dilution of 1:1000.

### Immunoprecipitation and in *vitro* cleavage assays

Stable HEK 293 cell lines overexpressing DICER1 mutants were generated by gamma-retroviral infection produced by HEK293T cells that were transfected with pQCXIB-1xFLAG-DICER1, pUMVC and pVSVG plasmids. Stable cell lines were then grown to confluency in a 15 cm dish and pelleted. Frozen pellets were then lysed with 500ul of NP-40 lysis buffer (20 mM Tris–HCl, pH 7.6, 1mM EDTA, 150 mM NaCl, 0.4% (v/v) NP-40 (Igepal CA630) and complete protease inhibitor (Roche)). Pellets were lysed at 4°C for 1 h. Lysates were then centrifuged at max speed for 15 min. The pre-cleared lysate was then incubated with 60 μl of FLAG-M2 resin (Sigma) or agarose beads (Sigma), as a negative control, O/N at 4°C on a rotator. The following day, the resin was washed twice with NP-40 lysis, twice with IP wash buffer (20 mM Tris–HCl, pH 7.6, 1 mM EDTA, 300 mM NaCl, 0.4% (v/v) NP-40), and twice with DICER1 cleavage buffer (20 mM Tris–HCl, pH 7.6, 50 mM NaCl, 20% glycerol, protein inhibitor (1:7)). Finally, DICER1 was eluted with 0.5 μg/μl of 3× FLAG peptide (Sigma) in DICER1 cleavage buffer. *In vitro* cleavage assays were previously described (18), which allows recognition of the pre-miRNA, and 5p/3p miRNA cleavage products on the same gel as the 5p miRNA is designed to be longer than the 3p miRNA. To generate pre-miR-122, a three primer PCR was done using pre-miR-122 primers. The PCR product was then purified using the QIAquick Gel Extraction kit (Qiagen) as per manufacturer's protocol. Pre-miRNA was transcribed via T7 polymerase with the MAXI-script kit using 200 ng of pre-miR-122 template and 6 μl of radioactive UTP ([α (32)_P]-800 ci/mmol) (PerkinElmer). The reaction was incubated at 37°C for 2 h. Then 1 μl of DNase was added to the reaction that was then incubated at 37°C for one extra hour. For each variant tested, immunoprecipitated FLAG-DICER1 was incubated with radiolabeled pre-miRNA in DICER1 cleavage buffer (see above) and 25 mM MgCl_2_, 20 mM DTT, and 0.25 μl of RNase inhibitor (TakaraBio) for a total volume of 20 μl. The reaction was incubated at 37°C and stopped at different intervals, including at the beginning of the reaction, after 1, 2 and 3 h by the addition of 20 μl of RNA gel loading dye (Thermo Fisher Scientific) and then placed on ice.

Cleavage products were resolved on a 7 M urea, 15% polyacrylamide gel (19:1; acrylamide: bisacrylamide) dissolved in 1× tris–borate–EDTA (TBE). The gels were then wrapped in saran wrap and exposed to a phosphor screen for 24 h at 4°C. The screen was then imaged by a phosphorimager (GE Storm 840, GE Healthcare or Amersham TYPHOON, Cytiva). Quantification of images was done by calculating the percent of cleaved product as previously described for DICER1 cleavage assays using ImageJ (19).

### Competition assays

Conditions were the same as for the *in vitro* cleavage assay except that, in addition to 50 ng of WT DICER1 protein, 50, 100 and 300 ng of competing protein was added to the reactions. Also, the cleavage reactions were only incubated at 37°C for 1 h.

### EMSA

Pre-let-7i, and pre-miR-100 were generated the same way as pre-miR-122 was for the *in vitro* cleavage reaction, with the exception that different primers were used for generation of the pre-let-7i and pr-miR-100 templates. 20 ng of radiolabelled RNA was then added with increasing amounts of DICER1 protein (0, 50, 100, 300 ng) in a binding solution consisting of 1 mM EDTA, 10 mM DTT, 20 mM Tris, 20% glycerol and 4.375 μl of of Recombinant RNase Inhibitor (Takara) for a total volume of 35 μl. The mixture was then incubated on ice for 30 min. EDTA was added to the reaction to prevent DICER1 from cleaving the pre-miRNA while still allowing binding (20). 5 μl of EMSA loading buffer (20 mM tris, 50% glycerol, 0.03% bromophenol blue, 1 mM EDTA) was then added to the binding mixture and 30 μl of the resulting solution was loaded on a native 5% polyacrylamide TBE gel (29:1; acrylamide:bisacrylamide). The gel was then wrapped in saran wrap and exposed to a phosphor screen for 24 h at 4°C. The screen was then imaged by a phosphorimager (Amersham TYPHOON, Cytiva) and quantified using ImageJ.

### MiRNA profiling

*DICER1* knock-in mMSCs were generated following the same protocol used for infection of HEK 293 cells. Following one week of selection in 5 μg/ml of blasticidin containing alpha-MEM media, the cells were single sorted in a 96-well plate and expanded in 10 cm plates. Cells were allowed to expand between 90 and 100% confluency in 15 cm dishes. Cells were then directly lysed in the 15cm dish using the mirVana miRNA isolation kit (ThermoFisher Scientific) as per manufacturer's protocol. The miRNA was then diluted between 300 and 500 ng/μl. Murine miRNAs were measured using the NanoString nCounter analysis system as per manufacturer's protocol.

### Dual luciferase assays

Cells grown to 70% confluency in a 6-well plate were co-transfected with 20 ng of firefly plasmid and 20 ng of *Renilla* plasmid. Twenty-four hours post-transfection, the cells were harvested and lysed in Passive Lysis Buffer (Promega). The activity levels of the Renilla and firefly reporters were measuring using Dual-Luciferase Assay (Promega).

### DNA extraction and sequencing of *DICER1* hotspot mutation

The multinodular goiter formalin-fixed paraffin embedded (FFPE) block was sliced at 10 μm thickness and micro-dissected with a surgical blade based on the pathologist review of the biopsy. The scraped tissue was used for DNA extraction using the QIAmp DNA FFPE Tissue kit (Qiagen) as per manufacturer's protocol. DNA was amplified using a series of primers designed to amplify *DICER1* hotspots for FFPE DNA. If bands were observed, the resulting PCR was sent for Sanger sequencing by Genome Québec using the same primers that were used in the PCR.

### DNA extraction from patient saliva/blood and germline sequencing

5 ml of patient blood or 4 ml of patient saliva was used for germline sequencing. DNA was then extracted using the Gentra Puregene DNA kit (Qiagen) as per manufacturer's protocol for either blood or saliva. DNA was amplified and sequenced with the same protocol as for FFPE DNA.

### RNA extraction from patient blood and reverse-transcription-PCR (RT-PCR)

5 ml of patient blood was drawn and stored in EDTA-tubes. 3 ml was then used to extract RNA using the RiboPure Blood kit (Thermo Fisher Scientific) as per manufacturer's protocol. For cDNA generation, 1 μg of purified RNA was used with 200 ng of random hexamer primers, with 1 μl of 10 mM dNTP mix for a total volume of 13 μl. The mixture was then incubated at 65°C for 5 minutes and placed on ice for at least 1 minute. 4 μl of 5× First strand buffer, 1 μl of 0.1M DTT, 1 μl of RNase OUT Recombinant RNase Inhibitor, and 1μl of Superscript IV Reverse Transcriptase (Thermo Fisher Scientific) were then added to the mixture. The reaction was then incubated at 25 °C for 5 min, 50°C for 60 min and then inactivated at 70°C for 15 min. 2 μl of the cDNA was then used in a PCR with the same protocol used to amplify FFPE DNA and the amplicons were sequenced using Sanger sequencing (Genome Québec).

### Structural analyses

The DICER1 Platform/PAZ crystal structure (PDB:4NH3) (8) was used to model the variants on PyMOL version 2.5.0. To create a hydrophobic model, residues leucine (L), valine (V), isoleucine (I), glycine (G), methionine (M), phenylalanine (F), alanine (A) were changed to red to depict hydrophobic amino acids and all other residues were changed to blue to show hydrophilic residues. Mutations were introduced to the structure using the Wizard Mutagenesis script.

### Western blotting

Samples were prepared with equal volume of 2× Laemmli buffer (4% SDS, 20% glycerol, 120 mM Tris–Cl (pH 6.8)) and boiled at 95°C for 5 minutes. Samples were then separated on an 8% polyacrylamide gel electrophoresis (PAGE) gel. The samples were migrated at 165 V for 2 h in running buffer (1 M glycine, 1% SDS, 0.1 M Tris–HCl). The protein was then transferred to a 0.45 μm nitrocellulose membrane (BioRad) in transfer buffer (1 M glycine, 0.1 M Tris–HCl, 10% methanol) at 30 V and 4°C O/N. After blocking the membranes with 5% milk + TBST, the membranes were left in primary antibody O/N at 4°C. The following day the membranes were washed and incubated with secondary antibody for 1 h at room temperature. The resulting membranes were visualized by being covered with enhanced chemiluminescence substrate (BioRad). The membranes were placed in a plastic sleeve and visualized with ImageQuant LAS 4000 biomolecular imager (GE Healthcare).

### Data analysis

Quantification of autoradiography images was done using ImageJ 1.53K, NIH, USA.

### Statistical analyses

For analysis of *in vitro* cleavage assay and luciferase assay, *P*-values were calculated by one-way or two-way ANOVA using Dunnett's method with Prism version 8.4.0. All graphs were created using Prism version 8.4.0. For miRNA profiling, normalization of miRNA reads was done using nSolver analysis software version 4 and as suggested by NanoString consultants by initially excluding miRNA reads in which the max read count across samples were less than 50 and then normalizing against miRNAs in which the coefficient variance (CV) was less than 20% that of the lowest miRNAs measured. Fold change was then measured compared to the human WT *DICER1* knock in cell line. Volcano plots were generated by ROSALIND® (https://rosalind.bio/), with a HyperScale architecture developed by ROSALIND, Inc. (San Diego, CA). The limma R library (21) was used to calculate fold changes. *T*-test was used for *P*-value calculations with the Benjamini–Hochberg methods for controlling false discovery rate.

## RESULTS

### Identifying a cluster of germline missense variants that may associate with DTPS

**Figure 1.**
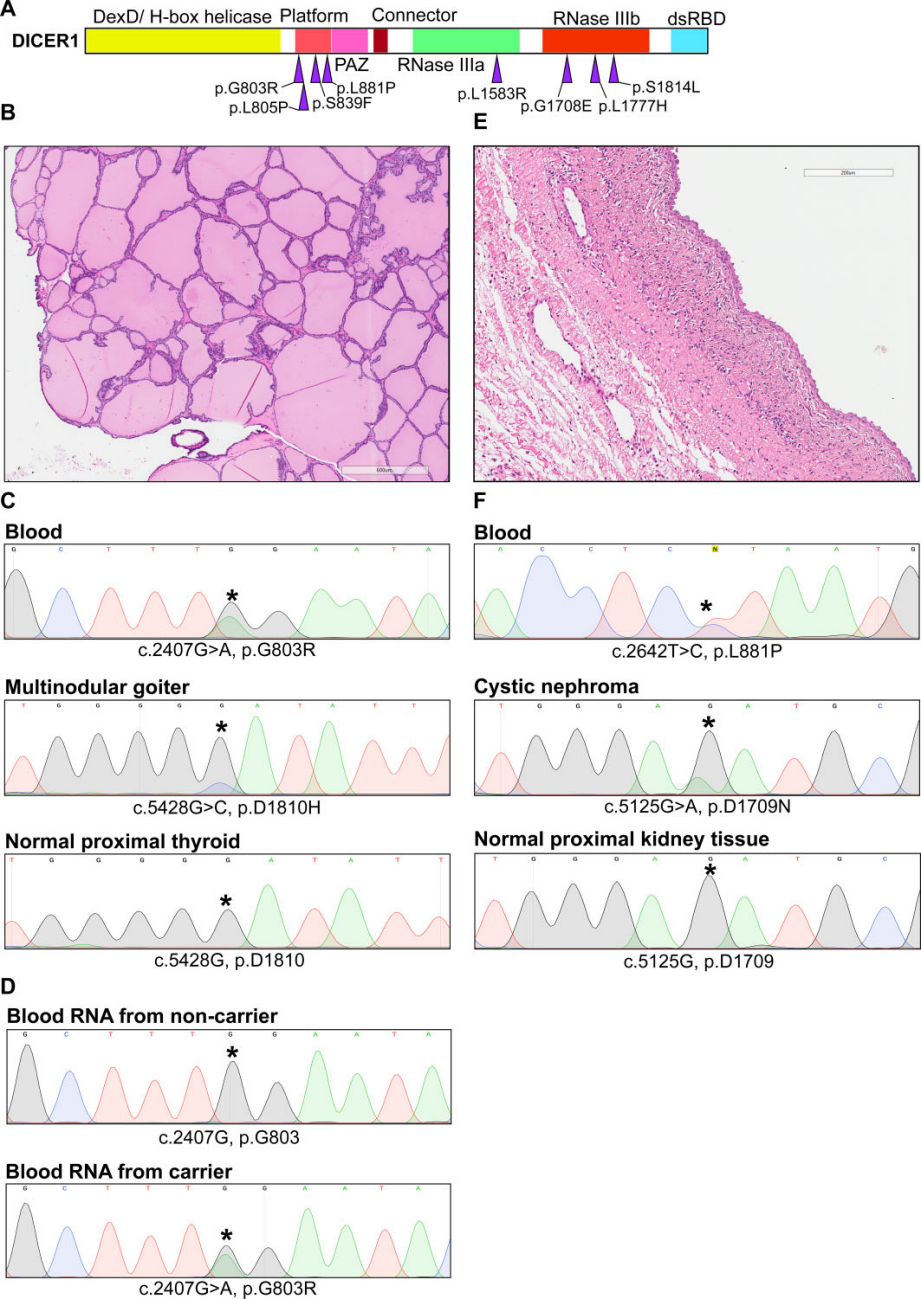
Clinical features of germline missense variants in Platform domain. (**A**) *DICER1* gene separated domains with germline missense variants associated with DICER1 tumor predisposition syndrome and with a population allele frequency under 0.01% and identified as pathogenic by 2/3 of our *in silico* predictors. (**B**) Hematoxylin and eosin staining of multinodular goiter showing follicles distended with colloid and lined by benign follicular epithelium. Small papillary infoldings are noted focally. 600 microns distance is represented by rectangle at bottom of image. Lesion from patient bearing germline *DICER1* c.2407G > A, p.G803R variant. (**C**) Sanger sequencing chromatograms of *DICER1* from blood, multinodular goiter, or normal proximal thyroid DNA from patient bearing *DICER1* c.2407G > A, p.G803R variant. Variants of interest are highlighted by an asterisk. (**D**) Sanger sequencing chromatograms from reverse-transcription polymerase chain reaction (PCR) from RNA extracted from lymphocytes of a non-carrier and carrier of the *DICER1* c.2407G>A, p.G803R variant. Variant of interest is highlighted by an asterisk. (**E**) Hematoxylin and eosin staining of cystic nephroma showing cyst lined by benign hobnail epithelium surrounded by collar of paucicellular mesenchyme. 200 microns distance is represented by rectangle at top of image. Lesion from patient bearing germline *DICER1* c.2642T>C, p.L881P variant. (**F**) Sanger sequencing chromatograms of *DICER1* from blood, cystic nephroma, or normal proximal kidney DNA from patient bearing germline *DICER1* c.2642T C, p.L881P variant. Variants of interest are highlighted by an asterisk.

We set out to gather data from the published literature on 18 germline *DICER1* missense variants seen in persons with clinicopathological manifestations that were mainly concordant with DTPS (Table 1). Within this list, we identified a cluster of missense variants that occurred in the Platform domain of *DICER1* (Figure 1A). To focus our efforts on variants that had a strong possibility of being disease-related, we filtered this list by only retaining variants with a population allele frequency in the genome aggregation database (gnomAD) under 0.01%, and that were predicted as damaging by at least two out of three *in silico* predictors: PolyPhen2, SIFT and Missense 3D (22,23). This analysis was done on all 18 variants and ultimately culminated with a list of four variants that clustered in the Platform domain (p.G803R, p.L805P, p.S839F and p.L881P), 1 in the RNase IIIa domain (p.L1583R), and 3 in the RNase IIIb domain (p.G1708E, p.L1777H, p.S1814L) (Figure 1A, Table 1). Given the unknown role of the Platform in DTPS, we decided to focus on variants in this domain. Based on our own laboratory data, we had previously indicated that we considered the variants p.G803R, p.L805P and p.L881P to be Likely Pathogenic, but we lacked further tumor sequencing information from persons with p.G803R and p.L881P germline variants. Additionally, from the variants in Table 1, three out of six cases in which second somatic *DICER1* mutations were identified in tumor DNA occurred in patients bearing germline missense variants in the Platform domain.

**Table 1.** List of germline *DICER1* missense variants in persons with DICER1 tumor predisposition syndrome (DTPS). List of missense variants in the *DICER1* gene identified in germline DNA of patients with phenotypes associated with DICER1 tumor predisposition syndrome (DTPS) compiled using data reported by (de Kock *et al.*, 2019). Variants functionally investigated in this study are in bold. Variants that had a population allele frequency under 0.01% and predicted to be pathogenic by 2/3 in silico predictors are underlined to show clustering of possibly pathogenic variants in platform domain. ^i^Second hit in euthyroid multinodular goiters of persons bearing the germline p.S839F variant was described in (31). ^ii^Second hit in differentiated thyroid carcinomas and multinodular goiter bearing the germline p.S1814L variant was described in (32). ^ Classified by the DICER1 Variant Curation Expert Panel (VCEP).# Likely Pathogenic based on our laboratory results (ClinVar entries: 933014, 690445). B: benign, CN: cystic nephroma, DTC: differentiated thyroid carcinoma, DTPS: DICER1 tumor predisposition syndrome, FH: favorable histology, LB: likely benign, LP: likely pathogenic, MNG: multinodular goiter, NOS: not otherwise specified, P: pathogenic, PPB: pleuropulmonary blastoma, PTC: papillary thyroid carcinoma, SLCT: Sertoli-Leydig cell tumor, VUS: variant of unknown significance, WES: whole exome sequencing, WGS: Whole genome sequencing, WT: Wilms tumor

#	Reference	Phenotypes	Protein change	cDNA change	Domain	Second somatic pathogenic variant found in tumor	Lesion with second hit	ClinVar classification	gnomAD allele frequency (%)	PolyPhen2 Prediction	Sift Prediction	Missense 3D Prediction
1	Zhang J, 2015 PMID: 26580448	Rhabdo-myosarcoma	p.V62I	c.184G>A	DExD/H-box helicase	Not done	Not done	LB/VUS	7.96E-04	Benign	Tolerated	Neutral
2	Walz AL, 2015 PMID: 25670082	WT-FH	p.I85M	c.255C>G	DExD/H-box helicase	Not found via WES and SNP array	None	LB	1.71E-02	Benign	Tolerated	Neutral
3	Gadd S, 2017 PMID: 28825729	WT-FH	p.G162D	c.485G>A	DExD/H-box helicase	Not found via WES and SNP array	None	B/LB	7.26E-02	Benign	Tolerated	Damaging: disallowed phi/psi
4	Wasserman JD, 2018 PMID: 29474644	PTC	p.P375R	c.1124C>G	DExD/H-box helicase	c.5428G > T, p.D1810Y	PTC	B/LB/VUS	3.29E-02	Probably damaging	Not tolerated	Neutral
5	Brenneman M, 2015 PMID: 26925222	PPB	p.I582T	c.1745T>C	DExD/H-box helicase	Not specified whether looked for or not	None	VUS	8.02E-04	Probably damaging	Not tolerated	Neutral
**6**	**Palculict TB, 2016 PMID: 26566882**	**WT-NOS, MNG**	**p.G803R**	**c.2407G>A**	**Platform**	**Loss of heterozygosity**	**WT**	**LP#**	**Not present**	**Probably damaging**	**Not tolerated**	**Damaging: Clash, buried glycine change, buried charge introduction**
**7**	**Diets IJ, 2018 PMID: 29351919**	**cERMS**	**p.L805P**	**c.2414T>C**	**Platform**	**c.5439G > T, p.E1813D**	**cERMS**	**LP#**	**Not present**	**Probably damaging**	**Not tolerated**	**Damaging: Proline introduced, disallowed phi/psi, buried/ exposed introduction**
**8**	**Rio Frio T, 2011 PMID: 21205968**	**MNG**	**p.S839F**	**c.2516C>T**	**Platform**	**c.5428G > T, p.D1810Y^i^; c.5439G > T, p.E1813D^i^**	**MNG**	**VUS/LP/P**	**Not present**	**Possibly damaging**	**Not tolerated**	**Neutral**
9	Wu MK, 2013 PMID: 23620094	WT-NOS	p.A872T	c.2614G>A	Platform	Not found via Sanger sequencing	None	B^	8.11E-02	Probably damaging	Tolerated	Neutral
**10**	**Van Engelen K, 2017 PMID: 28960912**	**Cystic nephroma, pineal cyst, cystic lung lesion, MNG**	**p.L881P**	**c.2642T>C**	**Platform**	**Not done**	**Not done**	**LP^#**	**Not present**	**Probably damaging**	**Not tolerated**	**Damaging: Buried proline introduced, disallowed phi/psi**
11	Walz AL, 2015 PMID: 25670082	WT-FH	p.R1368C	c.4102C>T	RNase IIIa	Not found via WES and SNP array	None	VUS, LB	3.54E-03	Possibly damaging	Tolerated	Neutral
12	Hill DA, 2009 PMID: 19556464	PPB	p.L1583R	c.4748T>G	RNase IIIa	Not done	Not done	LP^	N/P	Probably damaging	Not tolerated	Damaging: Buried hydrophilic introduced, buried charge introduced
13	Zhang J, 2015 PMID: 26580448	Rhabdo-myosarcoma	p.R1630C	c.4888C>T	Between RNase IIIa and IIIb	Not done	Not done	VUS/LB	1.77E-03	Benign	Tolerated	N/A
14	Brenneman M, 2015 PMID: 26925222	PPB	p.G1708E	c.5123G>A	RNase IIIb	c.5438A > G, p.E1813G	PPB	LP/P	Not present	Probably damaging	Not tolerated	Damaging: Buried charge introduced, buried glycine replaced
15	Wu MK, 2013 PMID: 23620094	WT-NOS	p.L1777H	c.5330T>A	RNase IIIb	Not found via Sanger sequencing	None	LB/VUS	2.93E-05	Probably damaging	Tolerated	Neutral
16	Wu MK, 2016 PMID: 26545620	DTC, SLCT, MNG, CN	p.S1814L	c.5441C>T	RNase IIIb	c.5126A > G, p.D1709G^ii^; c.5425G > A, p.G1809R^ii^; c.5428G > C, p.D1810H^ii^	DTC, MNG, SLCT	P^	Not present	Probably damaging	Not tolerated	Damaging: Buried H-bond breakage
17	Brenneman M, 2015 PMID: 26925222	PPB	p.D1822V	c.5465A>T	RNase IIIb	Not specified whether looked for or not	None	LP^	N/P	Probably damaging	Not tolerated	Neutral
18	Wegert J, 2015 PMID: 25670083	WT-NOS	p.G1886R	c.5656G>A	dsRBD	Not found via WGS	None	VUS^	N/P	Probably damaging	Tolerated	Neutral

Sanger sequencing of DNA extracted from formalin-fixed paraffin embedded (FFPE) thyroid nodules from individual V-8, (Figure 1B, Table 2, Supplementary Figure S1) heterozygous for the germline c. 2407G > A, p.G803R variant (Figure 1C, top chromatogram) revealed the c.5428G > C, p.D1810H hotspot pathogenic variant, situated within the RNase IIIb domain of *DICER1* (Figure 1C, middle chromatogram). The variant was identified in triplicate in each sequencing direction and was not present in normal proximal thyroid tissue (Figure 1C, bottom chromatogram). Reverse-transcription (RT)-PCR of RNA from lymphocytes of individual V-6 (non-carrier, Figure 1D, top chromatogram) and individual V-5 (carrier, Figure 1D, bottom chromatogram) from the same family (Table 2, Supplementary Figure S1) and subsequent Sanger sequencing revealed a heterozygous peak on the chromatogram. This analysis therefore indicates that RNA from the c.2407G > A, p.G803R allele is expressed. Similarly, we obtained FFPE sections from a cystic nephroma (Figure 1E) arising in a person with the c.2642T > C, p.L881P germline variant (Figure 1F, top chromatogram). DNA sequencing identified a different RNase IIIb hotspot variant (c.5125G > A, p.D1709N) in this tumor (Figure 1F, middle chromatogram) that was not present in nearby histologically normal kidney (Figure 1F, bottom chromatogram). Taken together with previously available tumor sequencing data for p. S839F (24) and p. L805P (25) these results strongly suggest that these four Platform germline variants are causal for DTPS-related tumors.

**Table 2.** Summary of clinical information from families affected by germline *DICER1* Platform missense variants. Clinical information from reports of germline pathogenic missense variants in the platform domain of DICER1 with additional information provided in this study. Tumors that have been resected and pathology has been confirmed are indicated in bold. Question mark uncertainty of the age of diagnosis. cERMS: cervical embryonal rhabdomyosarcoma; Dx: diagnosis; MNG: multinodular goiter; N/A: not available

Germline variant (original publication)	Individual	Sex	DICER1-associated disease status (age in years at dx)	Mutation status	Second hit
DICER1 c.2407G>A, p.G803R (Palculict et al., 2016, with additional clinical information provided in this study)	III-2	F	Thyroidectomy (18)	Present	Not known
	IV-2	F	Unaffected	Absent	Not applicable
	IV-4	F	Unaffected	Absent	Not applicable
	IV-5	M	Unaffected	Present	Not applicable
	IV-7	F	Wilms tumor (8)	Present	Wilms tumor: Loss of heterozygosity (LOH) (patient tested unspecified)
	IV-8	M	Wilms tumor (4)	Present	
	V-5	M	**Wilms tumor** (4)	Present	
	V-6	F	Unaffected	Absent	
	V-8	F	**Wilms tumor** (2) **Thyroid nodular hyperplasia** (16)	Present	In this study: MNG: c.5428G>C, p.D1810H
	VI-1	M	Unaffected	Absent	Not applicable
DICER1 c.2414T>C, p.L805P (Diets et al., 2018)	I-1	M	Unaffected	Present	Not applicable
	I-2	F	Unaffected	Absent	Not applicable
	II-1	F	**cERMS** (4)	Present	cERMS: c.5439G>T, p.E1813D
DICER1 c.2516C>T, p.S839F (Rio Frio et al., 2011)	See table 1D from Rio Frio et al., 2011, for information regarding the 20 cases of MNG diagnosed in persons carrying with the p.S839F variant				MNG: c.5428G>T, p.D1810Y and c.5439G>T, p.E1813D (de Kock *et al.*, 2016)
DICER1 c.2642T>C, p.L881P (Van Engelen et al., 2017 and second hit provided in this study)	I-1	N/A	MNG (?)	N/A	Not known
	II-1	N/A	Goiter (?)	N/A	Not known
	II-2	M	Unaffected	Present	Not applicable
	III-1	F	**Cystic nephroma (1.1)** Pineal cyst (3.8)	Present	In this study: cystic nephroma: c.5125G>A, p.D1709N

### Select *DICER1* platform mutants display impaired pre-miRNA cleavage capacity *in vitro*

**Figure 2.**
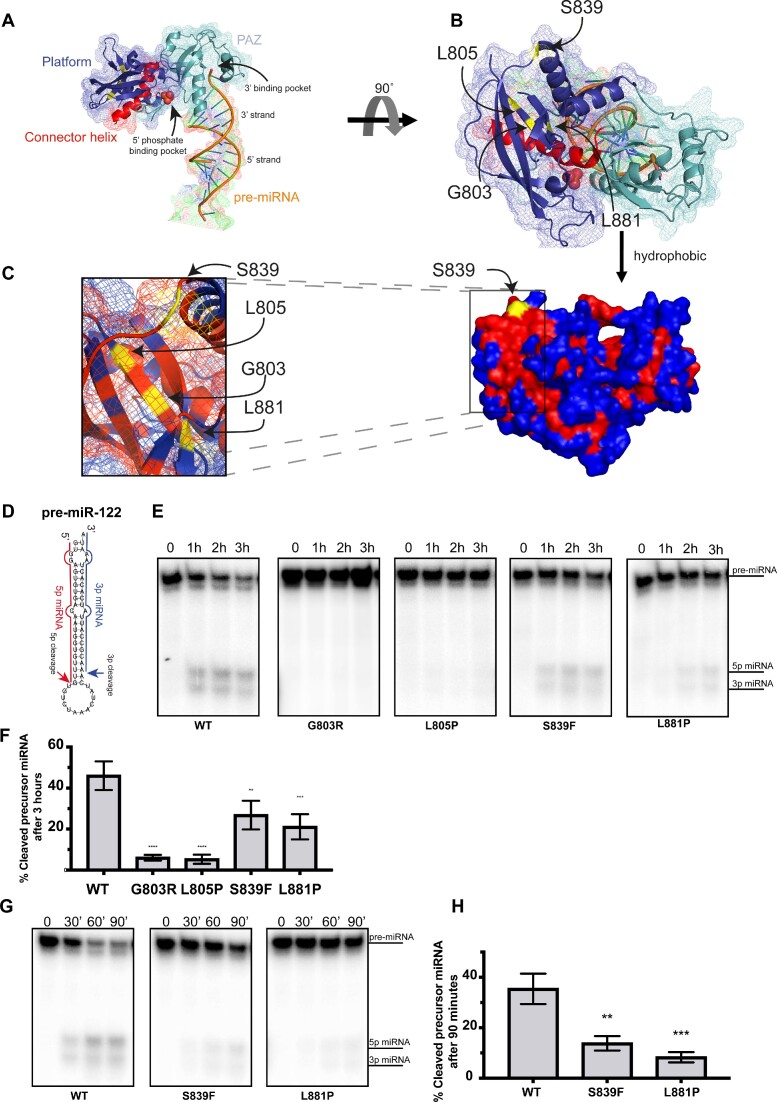
Positioning and impact of Platform variants on DICER1-mediated pre-miRNA cleavage. (**A**) Structure of platform (dark blue) and PAZ (cyan) domains with connector helix (red) on crystal structure published by (Tian *et al.*, 2014) (PDB: 4NH3). 5′ phosphate and 3′ binding pocket indicated by arrows. Theoretical pre-miRNA 3′ and 5′ strands are labelled on dsRNA used in crystal structure. (**B**) Spatial location of platform variants (yellow). (**C**) Surface model with hydrophobic residues coloured in red and hydrophilic ones in blue. Pathogenic variants are highlighted in yellow. (**D**) Structure of pre-miR-122 used in *in vitro* cleavage assay with indicated cleavage events. (**E**) Auto-radiograph of *in vitro* cleavage (IVC) assay for DICER1 Platform variants over 1 h time intervals using pre-miR-122. (**F**) Quantification of the percentage of cleaved product for each IVC reaction after 3 h compared to WT DICER1 (C) (*n* = 3). (**G**) Auto-radiograph of *in vitro* cleavage (IVC) assay for DICER1 Platform mutants over 30 min time intervals. (**H**) Quantification of the percentage of cleaved product for each IVC reaction compared to WT DICER1 after 90 min (D) (*n* = 3). Data information: In (D, F), data are presented as mean ± SD. *: 0.05 ≥ *P*-value > 0.01, **: 0.01 ≥ *P*-value > 0.001, ***: 0.001 ≥ *P*-value > 0.0001, ****: 0.0001 ≥ *P*-value (one-way ANOVA with Dunnett's multiple comparisons test compared to WT DICER1 cleavage).

Given that the segregation and tumor sequencing data reported above lends credence to the notion that these variants are clinically important, it is interesting that an analysis of the *DICER1* Platform domain crystal structure (Figure 2A, B) revealed that p.G803, p.L805 and p.L881, are located near each other in a hydrophobic core (with p.S839 being close to this core) but not in the 5′ phosphate binding pocket (Figure 2C). Nevertheless, based on these data we hypothesized that germline variants in these residues may impact DICER1 activity. To test this, we carried out *in vitro* cleavage experiments with various DICER1 mutants. Briefly, a human pre-miR-122 (Figure 2D) internally labelled with (32P-UTP, was incubated with FLAG-tagged wild-type or mutant DICER1 proteins that were expressed and immunopurified from HEK-293 with FLAG antibody (Supplementary Figure S2). Pre-miR-122 cleavage by each FLAG-tagged DICER1 protein was then assessed by denaturing polyacrylamide gel electrophoresis (PAGE) followed by autoradiography. Wild-type DICER1 efficiently cleaved the miRNA precursor, leading to the production of both 5p and 3p miRNAs after 1 h of incubation (Figures 2E and F). In contrast, two *DICER1* germline Platform mutants, p.G803R and p.L805P, failed to cleave pre-miR-122 even after 3 h of incubation *in vitro* (Figures 2E and F). Two other *DICER1* Platform domain mutants, p.S839F and p.L881P, produced mature miRNAs albeit at significantly lower levels as compared to DICER1^WT^ (Figures 2E and F). While p.S839F and p.L881P mutants were partially defective to differing degrees by 3 hrs incubation with pre-miR-122, incubating these *DICER1* variants with a pre-miRNA for shorter periods of time (0.5, 1 and 1.5 h) further demonstrated their impaired cleavage capacities as compared to wild-type DICER1 protein (Figure 2G and H). Taken together, these results suggest that several patient-derived *DICER1* Platform domain variants fail to cleave a pre-miRNA *in vitro*.

### Platform *DICER1* variants fail to support miRNA expression and miRNA-mediated gene silencing in cells

**Figure 3.**
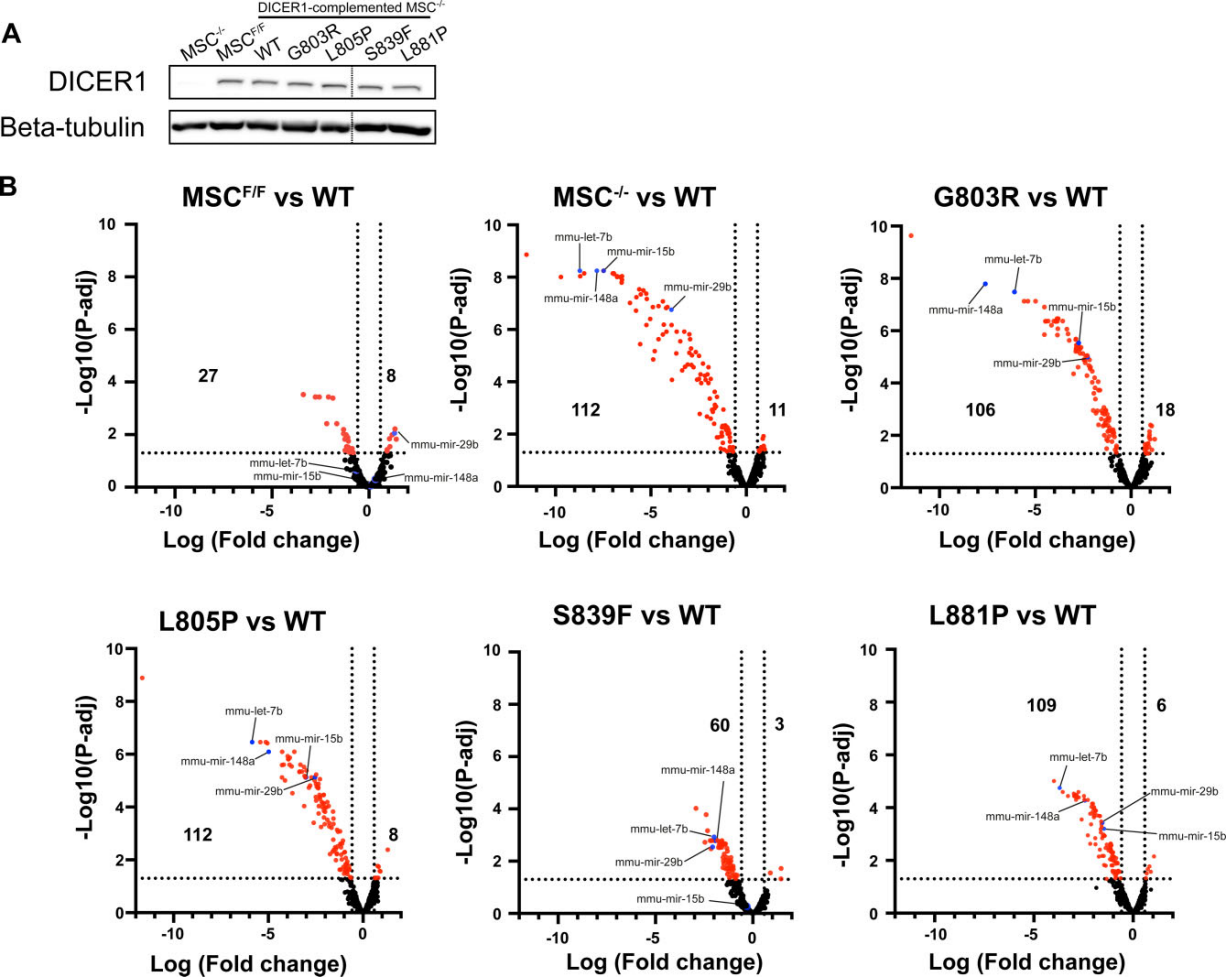
MiRNA profiling of murine mesenchymal stem cells (mMSCs). (**A**) Western blot of DICER1 protein in mMSCs expressing different DICER1 mutant proteins. (**B**) Volcano plots depicting differential expression of miRNAs between various cell line compared to WT. Significantly differentiated miRNAs are shown in red. miRNAs used in luciferase assay are shown in blue. Number of significantly differentiated miRNAs are shown on the left (downregulated) or right (upregulated) (*n* = 3). Data information: In (B), vertical doted lines represent fold change equal to –1.5 or 1.5. Horizontal doted line represents *P*-adjusted equal to 0.05 (*n* = 3).

Our *in vitro* cleavage assays suggest that these germline *DICER1* variants inhibit miRNA processing. To determine the extent of these defects, we stably complemented *Dicer*1^−/−^ murine mesenchymal stem cells (MSCs) with *DICER1^WT^*, or *DICER1* germline variants (Figure 3A). miRNA populations were subsequently isolated and evaluated by NanoString miRNA Platform analysis (Figure 3B). While miRNA populations dramatically plummeted in *Dicer*1^−/−^ MSCs as compared to the *Dicer*1^Flox/Flox^ MSCs, exogenous expression of *DICER1^WT^* significantly rescued global miRNA levels. In contrast, MSCs rescued with germline *DICER1* Platform domain variants failed to rescue miRNA levels to differing degrees, with p.G803R, p.L805P, and p.L881P having the greatest impact on miRNA populations.

**Figure 4.**
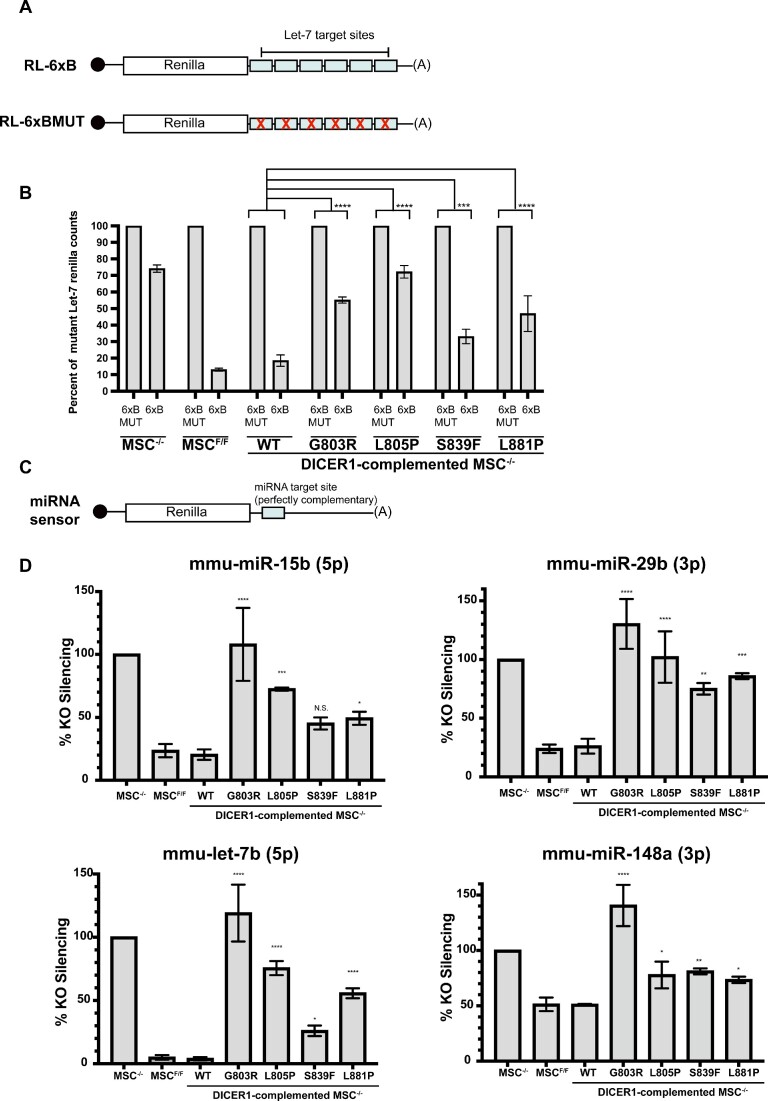
Luciferase assay validation of miRNA profiling data. (**A**) Schematic of luciferase assay used to measure levels of let-7 miRNAs. Top image depicts construct with let-7 targets sites in the 3′ untranslated region (UTR) of *Renilla* (RL-6xB). Bottom image depicts scenario in which *Renilla* mRNA is not targeted by let-7 miRNAs due to imperfect seed sequence hybridization (RL-6xBMUT). (**B**) Percent of *Renilla* silencing mediated by let-7 miRNAs using six equal let-7 seed sequence on *Renilla* mRNA 3′UTR (6xB) compared to basal *Renilla* expression measured using *Renilla* mRNA 3′UTR containing a mutated let-7 seed sequence (6xBMUT) (*n* = 3). (**C**) Schematic of reporter constructs used to measure levels of individual miRNAs using seed and complementary sequence hybridization that ultimately cause small interfering (si)RNA-induced silencing rather than miRNA-induced silencing. (**D**) Luciferase silencing for 4 miRNAs identified in (Figure 5B) using seed and complementary sequence hybridization. Percent *Renilla* silencing compared to DICER1 KO murine mesenchymal stem cell (mMSC) line (*n* = 3). Data information: In (B, D), data are presented as mean ± SD. *: 0.05 ≥ *P*-value > 0.01, **: 0.01 ≥ *P*-value > 0.001, ***: 0.001 ≥ *P*-value > 0.0001, ****: 0.0001 ≥ *P*-value (one-way ANOVA with Dunnett's multiple comparisons test compared to WT DICER1).

Our analysis indicates that miRNAs from the let-7 family were downregulated to differing degrees in our germline *DICER1* Platform domain variant rescued cell lines (Supplementary Figure S3). To determine whether alterations in miRNA levels associated with different germline *DICER1* variants impacts miRNA-mediated gene silencing, we transfected cells with plasmids encoding a *Renilla* luciferase (RL) reporter mRNA with six let-7 miRNA target sites (RL-6xB) or six mutated let-7 sites (RL-6xBMUT) in its 3 'UTR (Figure 4A). 48 hours post-transfection, cells were lysed and RL-6xB silencing was assessed using luciferase assays, with RL-6xBMUT serving as a control. RL-6xB was efficiently silenced in *Dicer1*^−/−^ MSCs complemented with exogenous *DICER1^WT^* (Figure 4B). In contrast, *Dicer1*^−/−^ MSCs complemented with *DICER1^G803R^* or *DICER1^L805P^* displayed significant defects in silencing the RL-6xB reporter mRNA as compared to *Dicer1*^−/−^ MSCs complemented with *DICER1^WT^*(Figure 4B). Similar results were observed when using other *Renilla* luciferase reporter mRNAs harboring single perfectly complementary sites to a number of different differentially expressed miRNAs, including mmu-miR-29b, mmur-miR-148a, and mmu-miR-15b (Figures 4C and D). Taken together, these data indicate that germline *DICER1* Platform domain variants significantly impact miRNA expression, and as a result fail to establish miRNA-mediated gene silencing in cells.

### Platform domain variants prevent DICER1 from binding to pre-miRNAs

**Figure 5.**
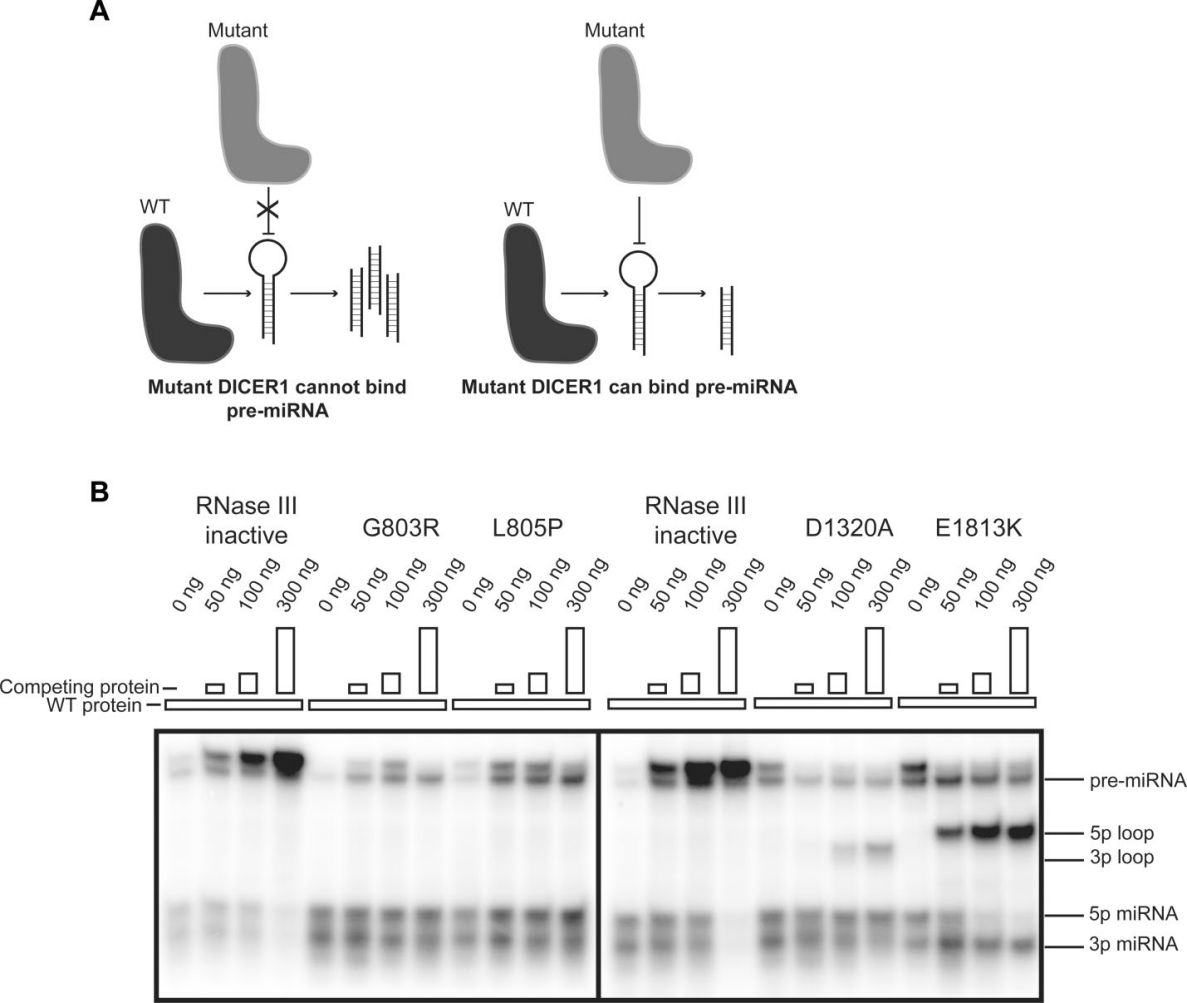
Competition assay of select DICER1 variants. (**A**) Schematic of expected results of competition assay based on whether DICER1 mutants can bind pre-miRNA or not. (**B**) Auto-radiograph of competition assay with increasing amounts of competing protein. RNase III inactive mutant represent DICER1 bearing the p.D1320A and p.E1813K mutations rendering the protein unable to cleave, but able to bind pre-miR-122.

The *in vitro* cleavage and miRNA profiling experiments suggested that several germline Platform domain variants render DICER1 unable to process pre-miRNAs. This could be due to these variants directly impacting the activity of the DICER1 RNase III domains such that they are not able to cleave pre-miRNAs. However, another plausible model would be that these variants render DICER1 unable to bind pre-miRNAs stem-loops (Figure 5A). In keeping with the latter model, adding increasing amounts of either DICER1^G803R^ or DICER1^L805P^ Platform domain variants failed to prevent DICER1^WT^ from processing a radiolabeled pre-miR-122 (Figure 5B). This is in contrast to competition assays whereby adding increasing amounts of an RNase-inactive DICER1 mutant, which harbors points mutations in both RNase IIIa (D1320A) and IIIb (E1813K) domains, gradually diminished the ability of DICER1^WT^ to generate 5p and 3p miRNAs (Figure 5B). Similarly, adding increasing amounts of an RNase IIIa- or IIIb- dead DICER1 variants led to a decrease in the ability of DICER1^WT^ to generate mature 3p and 5p miRNAs, respectively (Figure 5B).

**Figure 6.**
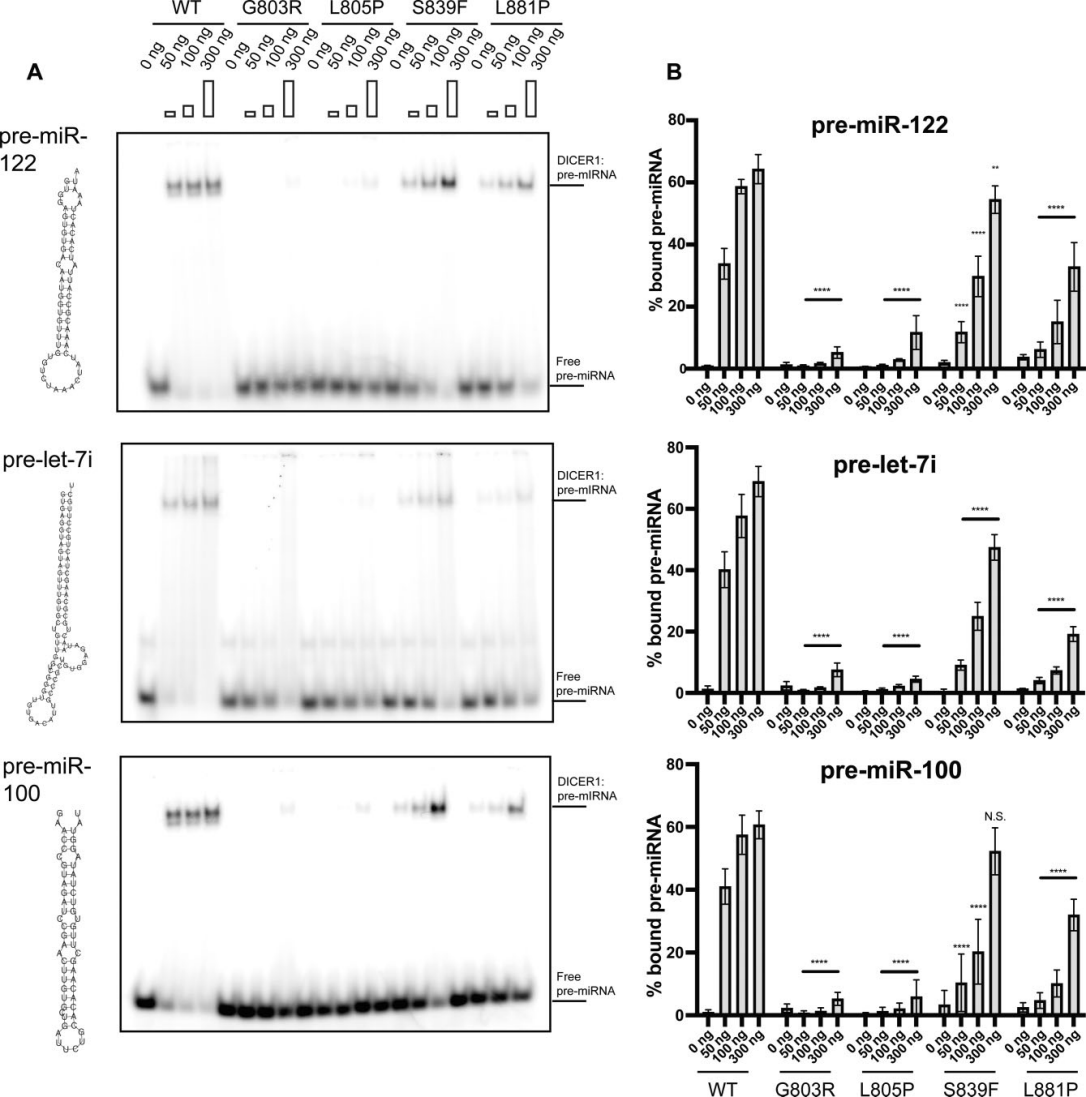
Electro-mobility shift assay (EMSA) of select DICER1 variants. (**A**) Auto-radiography of EMSA using three different pre-miRNAs with increasing amounts of DICER1 protein. (**B**) Quantification of corresponding EMSA (A) (*n* = 3). Data information: In (B), data are presented as mean ± SD. *: 0.05 ≥ *P*-value > 0.01, **: 0.01 ≥ *P*-value > 0.001, ***: 0.001 ≥ *P*-value > 0.0001, ****: 0.0001 ≥ *P*-value (two-way ANOVA with Dunnett's multiple comparisons test compared to WT DICER1).

To directly test if *DICER1* Platform domain variants are defective in pre-miRNA binding we next carried out electrophoretic mobility shift assays (EMSAs). Briefly, constant amounts of different radiolabelled pre-miRNAs (i.e. pre-miR-122, pre -let-7i and pre-miR-100) were titrated with increasing amounts of immunopurified DICER1^WT^ or select Platform domain variants. Following their incubation, RNA-protein complexes were resolved by native polyacrylamide gel electrophoresis (PAGE) and analyzed using a phosphorimager (Figures 6A and B). While DICER1^WT^ efficiently bound all pre-miRNAs tested, DICER1^G803R^ and DICER1^L805P^ failed to bind to pre-miRNA stem-loops. Moreover, other Platform domain variants (p.S839F and p.L881P) that were partially defective in generating mature miRNAs *in vitro* and in cells, failed to efficiently bind to pre-miRNAs *in vitro*. Taken together, these data indicate that germline DICER1 Platform domain variants are defective in pre-miRNA processing due to their inabilities to bind to pre-miRNA stem-loops.

## DISCUSSION

In this report, we have reviewed data from 18 germline missense *DICER1* variants and have identified four variants that are causal for DTPS. Using structural modeling, cell-free and cell-based assays we demonstrate that these missense variants structurally localize within the Platform domain and functionally behave as atypical loss of function variants.

**Figure 7.**
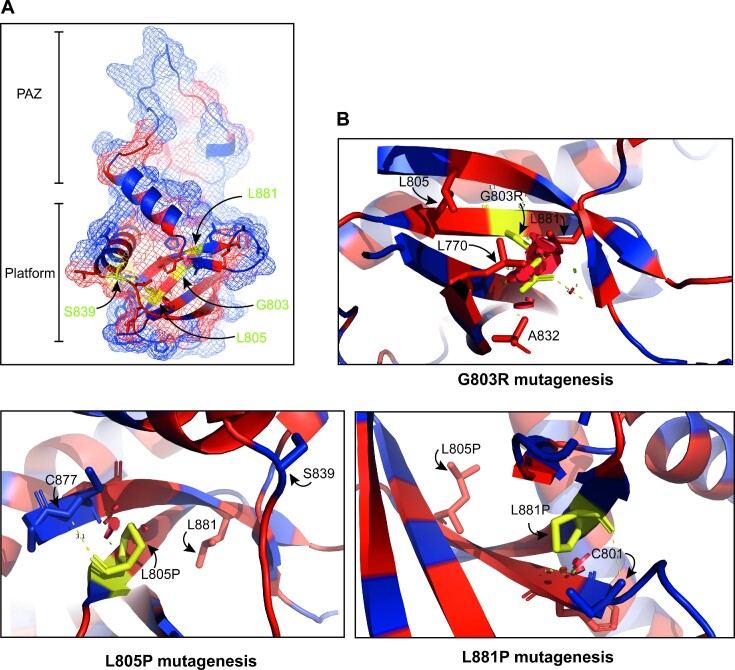
Structural consequences of DICER1 Platform variants. (**A**) Overview location of pathogenic variants (yellow) on Platform/PAZ crystal structure with hydrophobic residues in red and hydrophilic residues in blue (Tian *et al.*, 2014) (PDB: 4NH3). (**B**) Consequences of side chain substitution for the specific variants found in the hydrophobic core using the PyMol Wizard mutagenesis script. Clashes are depicted by red plates that represent van der Waals interactions. Other residues proximal to the clashes are listed in the panel.

The DICER1 Platform domain contains a 5′-recognition pocket that is required for DICER1 to recognize the pre-miRNA 5′-phosphate, with mutations in this basic motif reducing both cleavage efficiency and cleavage sites (7,8). We report here on several germline DTPS-associated Platform domain variants that are defective in pre-miRNA processing. Intriguingly, these variants do not reside within the Platform domain 5′-phosphate pocket. Molecular modeling suggests that the p.G803R variant introduces a long basic side chain that would clash with nearby residues L770, A832 and L881 (Figure 7). In addition, the p.L805P and p.L881P variants, which introduce a proline in the middle of their beta sheets may alter the overall Platform domain structure by disrupting the hydrogen-bonding network. In keeping with this, while p.L881P impaired DICER1 activity, the germline p.L881V variant, which should still form hydrogen bonds similar to L881, did not affect DICER1 cleavage *in vitro* (data not shown). Nevertheless, exactly how these variants alter the DICER1 structure remains to be established.

Recently, the structural basis for DICER1-mediated pre-miRNA cleavage has been further elucidated. It was shown that the PAZ domain is reoriented to accommodate RNA for cleavage and that the Platform domain not only recognizes the 5′ phosphate of pre-miRNA, but also plays a role in determining the cleavage pattern of pre-miRNA based on their 5′ terminal base (11). Perhaps the flexibility of the PAZ domain may be impaired by these variants which in turn could explain the decreased substrate binding. Furthermore, it has been recently found that the recurrent DICER1 RNase IIIa variant (S1344L) impairs pre-miRNA cleavage similarly to somatic RNase IIIb variants by decreasing 5p miRNA levels (26,27). Interestingly, no change in 5p to 3p miRNA ratio has been seen with Platform variants, indicating a different mechanism of action in disrupting miRNA biogenesis.

**Figure 8.**
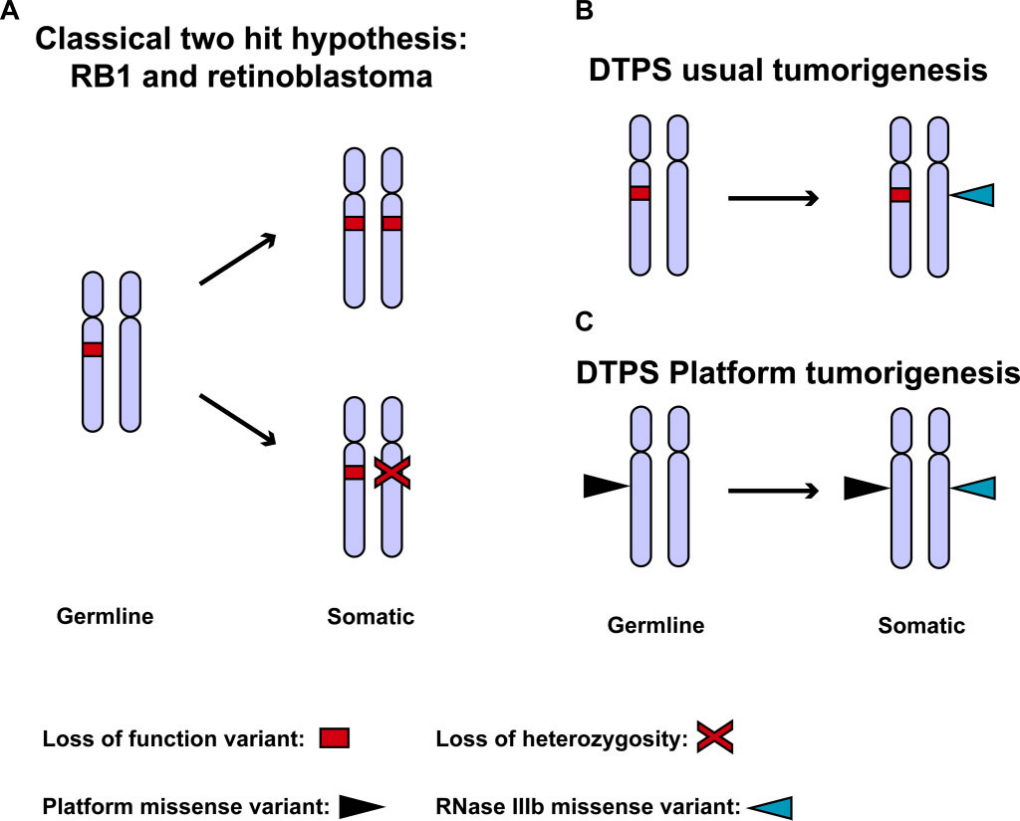
DICER1 tumor predisposition syndrome (DTPS) tumorigenesis. (**A**) Schematic of genetic events seen with classical tumor suppressor genes, for example *RB1*, during tumorigenesis. (**B**) Schematic of usual *DICER1* genetic events required for tumorigenesis in persons with DTPS. (**C**) Schematic of *DICER1* genetic events required for tumorigenesis in persons with germline Platform missense variants.

Most tumor suppressor genes associated with inherited cancer syndromes (e.g. retinoblastoma (28)) obey Knudson's two-hit rule (Knudson, 1993), whereby both the first (germline) PV and second (tumor-confined) hit usually result in loss of function of the affected allele, Figure 8A). DTPS is unusual because although the syndrome follows this two-hit model, the two hits are mechanistically distinct. In tumors occurring in DTPS, the first hit in *DICER1* is generally a protein-truncating nonsense variant, but the second hit is nearly always a missense variant affecting DICER1 RNase IIIb activity (12) (Figure 8B). Here, we describe a distinct DTPS phenomenon where the first hit is a missense rather than a nonsense variant (Figure 8C). However, as these missense variants in the Platform domain render DICER1 unable to bind pre-miRNA stem-loops, they are highly likely to be disease-associated alleles, and if occurring in a person with a tumor harboring a canonical RNase IIIb somatic mutant *in trans*, would be causal for DTPS. Nevertheless, it is generally held that *in vitro* data pertaining to the behavior of germline variants should not be used by itself as the basis of clinical decision-making (29). Indeed, despite the compelling data presented here, supported by recently published data studying some the same variants (30), when we applied rules from the American College of Medical Genetics (with specifications from the *DICER1* Variant Classification Expert Panel(VCEP) https://clinicalgenome.org/affiliation/50050/) in an attempt to re-classify these variants, the variants with clear defects in pre-miRNA stem-loop binding (p.G803R and p.L805P) remained classified as variants of uncertain significance (VUS) (Table 3). Therefore, until such time as the results of the binding and competition assays reported here are incorporated into *DICER1* variant classification rules, the results presented here can provide clinicians with a greater degree of confidence when interpreting rare variants in the Platform domain of *DICER1*.

**Table 3.** Classification of germline DICER1 Platform variants based on Variant Curation Expert Panel (VCEP) criteria. Classification of germline DICER1 Platform variants based on Variant Curation Expert Panel (VCEP) criteria based on functional and clinical data provided in this study and previous reports. cERMS: cervical embryonal rhabdomyosarcoma, MNG: multinodular goiter

	*DICER1* variant			
VCEP Criteria	**c.2407G>A, p.G803R** (Original reference: (Palculict et al., 2016) with additional information from this study)	**c.2414T>C, p.L805P** (Diets et al., 2018)	**c.2516C>T, p.S839F** (Rio Frio et al., 2011) (de Kock et al., 2014)	**c.2642T>C, p.L881P** (van Engelen et al., 2018)
PM2 Supporting: Allele frequency < 0.000005 across gnomAD with no more than one allele in any subpopulation and at least 20 x coverage.	PM2_Supporting: variant absent from gnomAD.	PM2_Supporting: Variant absent from gnomAD.	PM2_Supporting: Variant absent from gnomAD.	PM2_Supporting: Variant absent from gnomAD.
PP1 Supporting: 3–4 meioses across ≥ 1 family. Moderate: 5–6 meioses across ≥ 1 family.	PP1 not applied: 1 meiosis across 1 family.	PP1 not applied: No segregation data.	PP1_Moderate: 19 meioses across 1 family.	PP1_Supporting: 3 meioses across 1 family.
PP3 Supporting: For missense variants, REVEL score ≥ 0.75.	PP3_Supporting: REVEL score: 0.807.	PP3_Supporting: REVEL score: 0.944.	PP3 not applied: REVEL score: 0.519.	PP3_Supporting: REVEL score: 0.906.
PP4 Supporting: Somatic tumor testing identifies somatic hotspot second hit and no additional somatic LOF variants	PP4_Supporting: DICER1 c.5428G>C, p.D1810H in MNG.	PP4_Supporting: DICER1 c.5439G>T, p.E1813D in cERMS.	PP4_Supporting: DICER1 c.5428G>T, p.D1810Y and DICER1 c.5439G>T, p.E1813D in MNG.	PP4_Supporting: DICER1 c.5125G>A, p.D1709N in cystic nephroma.
PS3 Supporting: In vitro cleavage assay shows failure or severly reduced capacity to produce either 5p or 3p miRNAs from a pre-mIRNA	PS3_ Supporting: No cleavage.	PS3_Supporting: No cleavage.	PS3_Supporting: Impaired cleavage.	PS3_Supporting: Impaired cleavage.
PS4 Supporting: 1–1.5 phenotype points. Unrelated probands may contribute up to 1 point each.	PS4_Supporting: 1 phenotype point: Wilms tumor and MNG.	PS4_Supporting: 1 phenotype point: cERMS.	PS4_Supporting: 1 phenotype point: MNG.	PS4_Supporting: 1 phenotype point: Cystic nephroma, MNG, cystic lung lesion, pineal cyst.
Total points on modified Bayesian point system (variant classification)	5 (5 supporting criteria): Variant of uncertain significance.	5 (5 supporting criteria): Variant of uncertain significance.	6 (4 supporting and 1 moderate criteria): Likely pathogenic.	6 (6 supporting criteria): Likely pathogenic.

## DATA AVAILABILITY

The data underlying this article are available in the article and in its online supplementary material.

## Supplementary Material

zcad030_Supplemental_Files

## Notes

Present address: Mona Wu, Children's Hospital Los Angeles, University of Los Angeles, Los Angeles, CA, USA.
