# Supplementary material for: DICER1 platform domain missense variants inhibit miRNA biogenesis and lead to tumor susceptibility
Source: NAR Cancer. 2023 Jun 16;5(3):zcad030. doi: 10.1093/narcan/zcad030 (PMC10273190; doi:10.1093/narcan/zcad030)
Supplement: zcad030_Supplemental_Files [file zcad030_supplemental_files.zip › Supplementary Figures and text.pdf]

SUPPLEMENTARY FIGURE LEGENDS

Figure S1. Pedigree of family affected by *DICER1* c.5428G>C, p.G803R variant. Disease and carrier status of individuals affected by the *DICER1* c.5428G>C, p.G803R variant. Confirmed carrier status is indicated by +/-. Confirmed non-carrier status is indicated by +/+. Obligate carrier is indicated by [+/-]. Purple represents thyroid disease. Question mark refers to uncertain cause for the thyroidectomy. Beige represents Wilms tumor. Lesions that have been resected and pathology has been confirmed are indicated in bold. Dx: diagnosis, yo: years old.

Figure S2. Immunoprecipitation of FLAG-tagged DICER1 mutant protein. Western blot of FLAG-tagged DICER1 immunoprecipitation. Input represents total cell lysate, supernatant represents supernatant after beads were centrifuged, elution represent protein eluted by 3x FLAG peptide solution. Parental cell line represents HEK 293 cells not expressing exogenous DICER1 protein. Negative agarose beads are used as a negative control to show the absence of none-specific bead-binding.

Figure S3. Let-7 miRNAs expression across murine mesenchymal stem cells (mMSCs). Log2 normalized expression quantified by NanoString nCounter analysis for miRNAs part of the Let-7 family across murine mesenchymal stem cell (mMSC) lines) (n = 3).
Data information: Data are presented as mean ± SD.

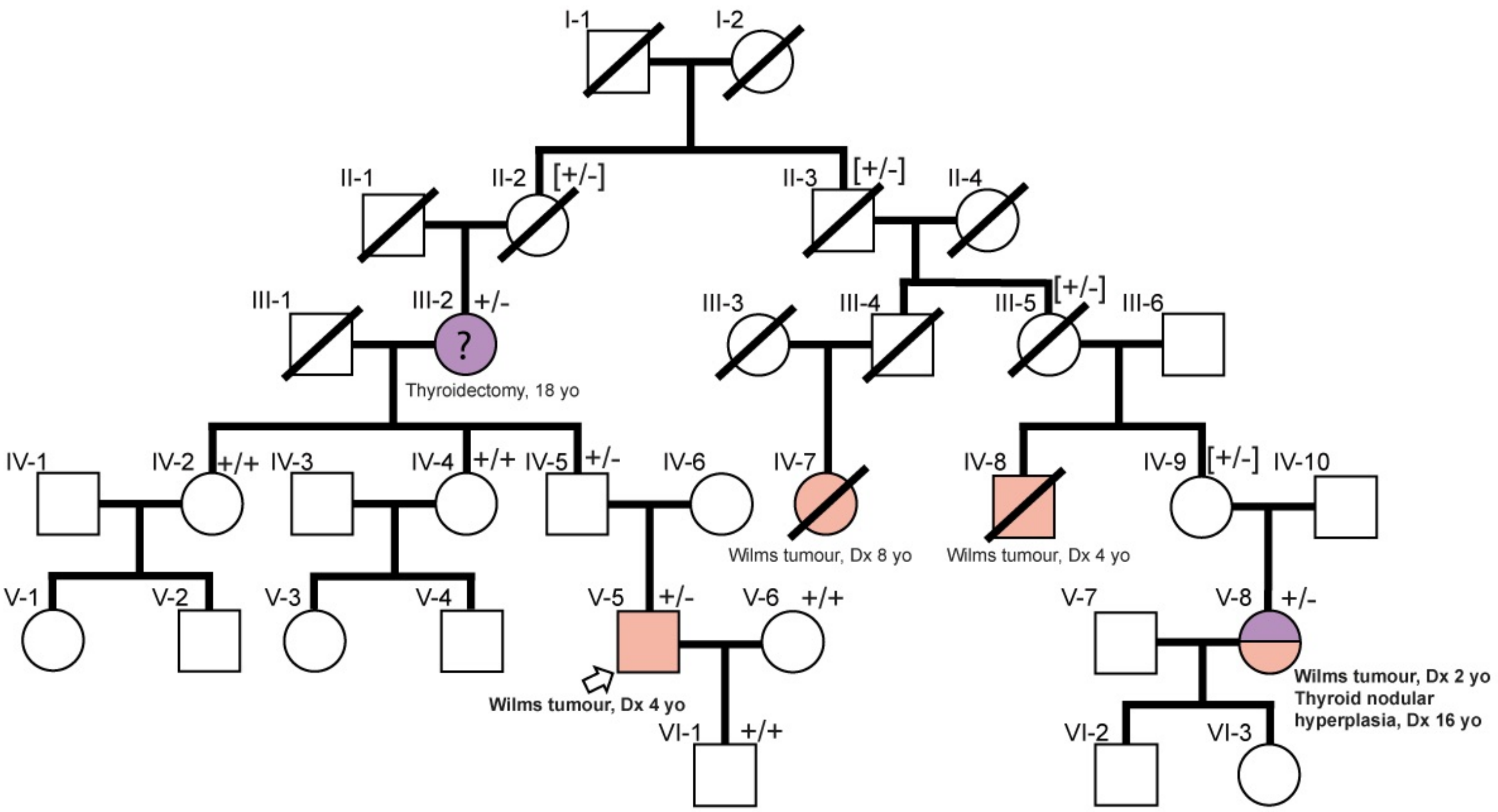

I-1
I-2
II-1
II-2
[+/-]
II-3
[+/-]
II-4
III-1
III-2
+/-
?
Thyroidectomy, 18 yo
III-3
III-4
III-5
[+/-]
III-6
IV-1
IV-2
+/+
IV-3
IV-4
+/+
IV-5
+/-
IV-6
IV-7
Wilms tumour, Dx 8 yo
IV-8
Wilms tumour, Dx 4 yo
IV-9
[+/-]
IV-10
V-1
V-2
V-3
V-4
V-5
+/-
V-6
+/+
Wilms tumour, Dx 4 yo
V-7
V-8
+/-
Wilms tumour, Dx 2 yo
Thyroid nodular
hyperplasia, Dx 16 yo
VI-1
+/+
VI-2
VI-3

Supp fig 1

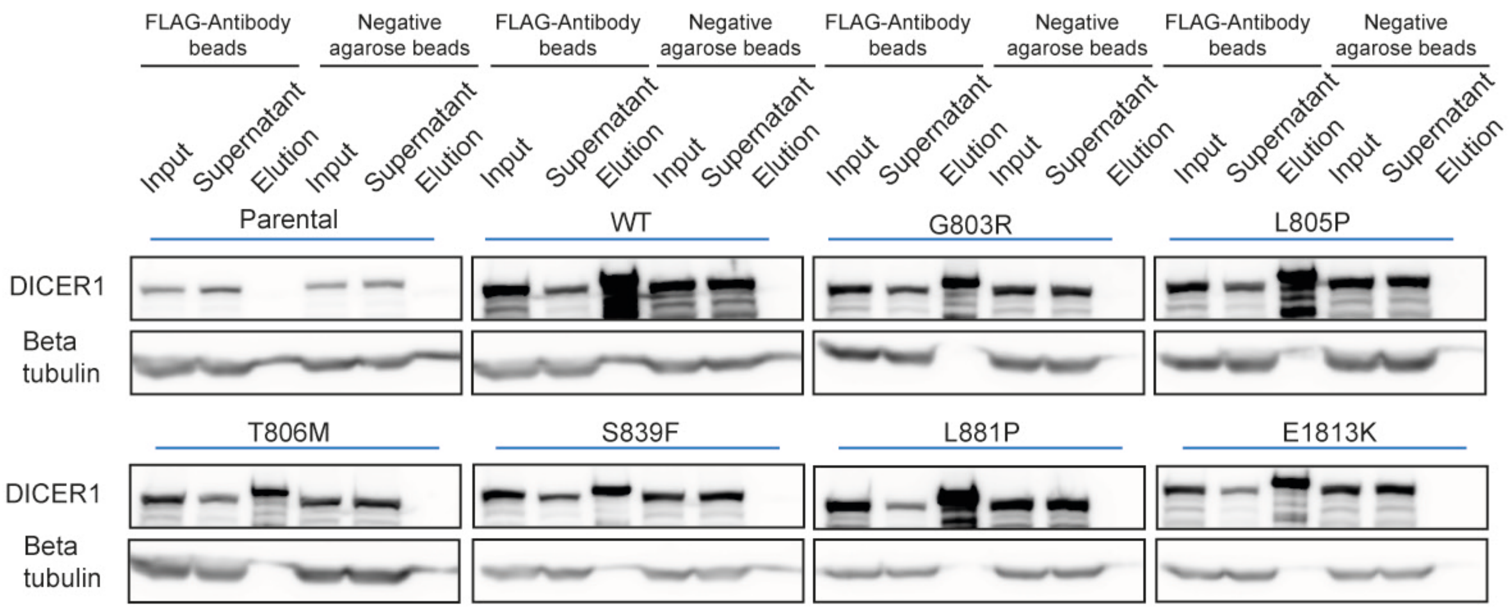
FLAG-Antibody beads
Negative agarose beads
Input
Supernatant
Elution
Parental
WT
G803R
L805P
T806M
S839F
L881P
E1813K
DICER1
Beta tubulin

Supp fig 2

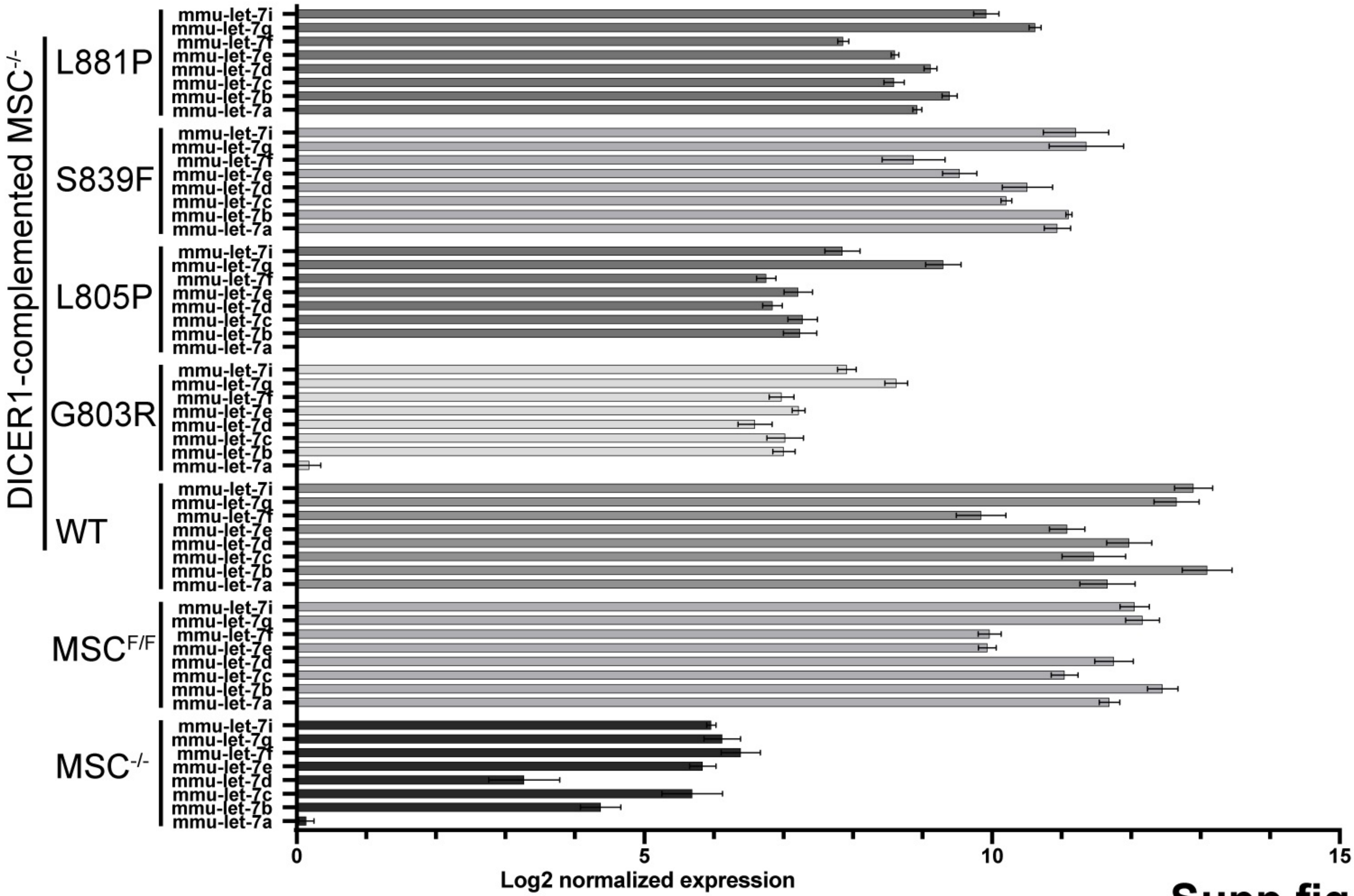
DICER1-complemented MSC-/-
L881P
S839F
L805P
G803R
WT
MSCF/F
MSC-/-
mmu-let-7i
mmu-let-7g
mmu-let-7f
mmu-let-7e
mmu-let-7d
mmu-let-7c
mmu-let-7b
mmu-let-7a
0
5
10
15
Log2 normalized expression

Supp fig 3
